# Spontaneous discontinuation of distressing auditory verbal hallucinations in a school-based sample of adolescents: a longitudinal study

**DOI:** 10.1007/s00787-019-01393-7

**Published:** 2019-08-27

**Authors:** Vera Brink, Catheleine van Driel, Saliha el Bouhaddani, Klaas J. Wardenaar, Lieke van Domburgh, Barbara Schaefer, Marije van Beilen, Agna A. Bartels-Velthuis, Wim Veling

**Affiliations:** 1 University of Groningen, University Medical Center Groningen, University Center for Psychiatry, PO Box 30.001 (HPC CC60), 9700 RB Groningen, The Netherlands; 2 Parnassia Institute, Carnissesingel 51, 3083 JA Rotterdam, The Netherlands; 3 Department of Psychiatry, Interdisciplinary Center Psychopathology and Emotion Regulation (ICPE), University of Groningen, University Medical Center Groningen, , PO Box 30.001, Groningen, 9700 RB The Netherlands; 4 Department of Child and Adolescent Psychiatry, VU University Medical Center, PO Box 303, 1115 ZG Duivendrecht, The Netherlands; 5 Department of Research and Development, Pluryn-Intermetzo, PO Box 53, 6500 AB Nijmegen, The Netherlands; 6 University of Groningen, University Medical Center Groningen, University Center for Psychiatry, Rob Giel Research center, PO Box 30.001, 9700 RB Groningen, The Netherlands

**Keywords:** Auditory verbal hallucinations, Adolescent, Psychopathology, Risk, Predictor, Distress

## Abstract

Auditory verbal hallucinations (AVH) can be transiently present in both clinical and healthy adolescent populations. It is not yet fully understood why AVH discontinue in some adolescents and persist in others. The aim of this explorative study is to investigate predictors of spontaneous discontinuation of distressing AVH in a school-based sample of adolescents. 1841 adolescents (mean age 12.4 years, 58% female) completed self-report questionnaires at baseline. The current study included 123 adolescents (7%; 63% female) who reported at least mild distressing AVH at baseline and completed follow-up measurements. LASSO analyses were used to uncover predictors of spontaneous discontinuation of distressing AVH. During follow-up, 43 adolescents (35%) reported having experienced distressing AVH during the last 12 months, while 80 adolescents did not. Spontaneous discontinuation of distressing AVH was predicted by never having used cannabis, parents not being divorced in the past year, never having been scared by seeing a deceased body, less prosocial behaviour, school grade repetition, having the feeling that others have it in for you, having anxiety when meeting new people, having lived through events exactly as if they happened before and having the feeling as if parts of the body have changed. No associations between spontaneous discontinuation of distressing AVH and age or ethnicity were found. Distressing AVH in non-clinical adolescents are mostly transient. Discontinuation was predicted up to a certain extent. However, several predictors were difficult to interpret and do not provide leads for preventive measures, except for discouraging cannabis use.

**Electronic supplementary material:**

The online version of this article (10.1007/s00787-019-01393-7) contains supplementary material, which is available to authorized users.

## Introduction

Auditory verbal hallucinations (AVH) are experienced by 5–35% of children and adolescents [1]. It is defined as the perception of hearing voices without actual auditory stimulation [2]. In most children and adolescents, AVH disappear spontaneously and do not cause functional impairment, but in less than 10% the AVH persist into adulthood [3–5]. Persistence of AVH is associated with an increased risk of developing severe psychopathology such as psychotic disorders [4, 6].

AVH are part of the group of psychotic experiences (PEs), which is a set of subclinical unusual perceptual experiences distributed throughout the general population [7]. Although PEs are subclinical experiences, they are associated with an increased proneness to psychotic disorders [7, 8]. A model that can be used to understand the role of PEs in relation to psychosis and other mental diseases is the psychosis proneness–persistence–impairment model [8, 9]. According to this model, the transition from experiencing AVH to, ultimately, developing a psychiatric disorder, is caused by exposure to a combination of environmental, genetic, demographic and psychological factors, that interact synergistically or additively with the AVH [7, 8, 10]. The phenomenology of AVH remains the same throughout the severity continuum, with non-clinical individuals at one end of the spectrum and individuals with a psychiatric disorder on the other end [8, 9].

A non-exhaustive summary of risk factors for AVH persistence in non-clinical adolescents includes: a concurrent depressed mood, delusional ideations, general psychopathology and adolescent instead of childhood onset of AVH [6, 11]. Ethnic minority status, cannabis use, childhood trauma, peer victimization, social and attentional problems, affective dysregulation, developmental problems and distress due to psychotic experiences are also associated with persistence of psychotic experiences in non-clinical adolescents, although not specific for AVH [12, 13].

Adding the dimension of distress to AVH persistence might help characterizing a group of voice hearing adolescents that is at risk of developing mental health problems. Most non-clinical individuals with AVH do not experience any distress from the AVH and do not need medical care for it [14].

However, some voice hearers do experience AVH-associated distress, which in turn is associated with help-seeking behaviour [6]. Importantly, Daalman and colleagues found that AVH-related distress in healthy adults was the most important predictor for transition to mental health problems at 5-year follow-up [14, 15]. Most other baseline characteristics, including age, family history of psychotic disorder, childhood trauma, and emotional valence, frequency and duration of AVH did not predict a need for mental health care at follow-up [15].

More research specifically on adolescents with AVH is needed, because the first prodromal symptoms of mental illness and associated social dysfunction often emerge during adolescence [6]. A reason for this might be that during adolescence a series of rapid changes in hormones and brain development, such as the reduction of connections in the brain by pruning, takes place [6, 16]. These changes and the associated stress can trigger psychopathology in some adolescents [17]. Next to the neurodevelopmental changes, emotional and social changes also take place during adolescence which might increase vulnerability to mental illness [6]. At the same time, attenuated psychotic symptoms (APS), such as AVH, might occur during early adolescence as a non-pathological expression of not yet fully matured cognitive abilities [18]. If risk factors or neurodevelopmental disturbances in information processing arise during the maturation process, the APS might persist and ultimately develop into psychiatric disorders [18].

### Current study

From a clinical perspective, an important question is thus why AVH disappear spontaneously in some adolescents, while in others the AVH persist, and are associated with distress and development of psychopathology. As persistence and distress of AVH are the most relevant determinants of help-seeking behaviour and future transition to mental illness, research should focus on adolescents with persistent AVH with associated voice hearing distress. When we know more about what promotes spontaneous discontinuation of distressing AVH, we might be able to stimulate protective factors timely and prevent persistence and possible subsequent development of psychiatric disorders. Therefore, the aim of the current longitudinal school-based study was to investigate what percentage of non-clinical adolescents experience AVH with distress, in how many adolescents AVH with distress persist, and which factors, in terms of demographic variables, psychological traits and environmental factors, are predictive of spontaneous discontinuation of distressing AVH. Based on previously found risk factors for AVH and general PE persistence in non-clinical adolescents, we hypothesized that the absence of delusional ideation, younger age, not being from an ethnic minority group, no use of cannabis and no experience of traumatic events would be associated with spontaneous discontinuation of distressing AVH.

## Materials and methods

### Participants and study design

**Fig. 1:**
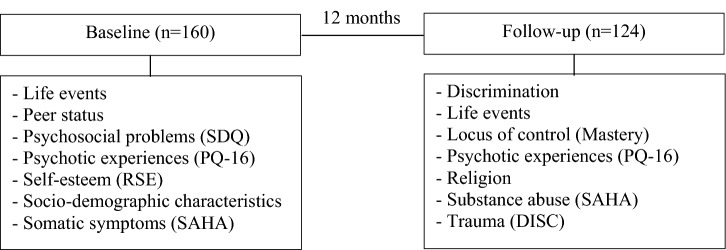
Overview of the questionnaires completed at baseline and follow-up

Data for this study was acquired from MasterMind, a large longitudinal cohort study on adolescent mental health in the general population [19]. In this study, data of 1841 adolescents from 12 secondary schools, with a minimal education level of secondary vocational education (VMBO), in the provinces Noord-Holland, Zuid-Holland, and Noord-Brabant in The Netherlands were collected from June 2013 until January 2015. Participants completed questionnaires in the first year of secondary school (baseline) and 1 year later, in the second year of secondary school (12-month follow-up). In Fig. 1, an overview of the measurements at each time point is shown. Approval for the MasterMind study was received from the Medical Ethics Committee of the VUmc [reference number 2013.247]. Parents received information about the study and an informed no-consent form, which they were requested to complete and return if they did not want their child to participate in the study or if their child did not want to. In that case the adolescent was excluded from the MasterMind study. Full descriptions of the MasterMind study procedure can be found elsewhere [19, 20].

For the current study, data was used from a subset of 160 participants who at baseline reported that they had experienced distressing AVH at some point in their life. The presence of distressing AVH was determined by a positive answer to the item “I have heard things other people cannot hear, like voices of people whispering or talking” on the Prodromal Questionnaire (PQ-16), in combination with experiencing at least mild distress associated with the AVH. The participants who did not complete both the baseline and the 12-month follow-up questionnaires were excluded from analyses.

### Definition of outcome

Outcome at 12-month follow-up was defined as spontaneous discontinuation of distressing AVH. The discontinuation group includes participants ‘who did not experience AVH anymore since baseline’ and ‘who did experience AVH since baseline, but without distress associated with the AVH’. The persistence group consists of participants ‘who experienced AVH with associated voice hearing distress since baseline’.

### Baseline measurements

At baseline, participants were asked to fill out questionnaires on socio-demographic characteristics. Ethnicity of the participants was determined by the ethnic background of themselves and their parents. The ethnicity was coded as migrant-Dutch if the participant, the father or the mother was born abroad. Education level was coded average when participants followed secondary vocational education (VMBO) and high when participants followed a higher secondary educational track (HAVO or VWO). Screening for psychotic experiences was done with the 16-item version of the Prodromal Questionnaire (PQ-16) [21]. The PQ-16 screens for subclinical psychotic symptoms and consists of 14 positive symptom items and 2 negative symptom items assessed on a two-point scale (0, not true; 1, true). The distress associated with the experiences is assessed on a four-point scale (0, no distress; 1, mild distress; 2, moderate distress; 3, severe distress). In this study, an experience was classified as a psychotic experience only if it was associated with at least mild distress. The question “I have heard things other people cannot hear, like voices of people whispering or talking” on the PQ-16 was used to screen for AVH. The Dutch PQ-16 as tested by De Jong et al. in a Dutch help-seeking population with a mean age of 14.6 years had a Cronbach alpha of 0.79 [22]. This questionnaire had good concurrent validity with both the interview-based Comprehensive Assessment of At-Risk Mental States (CAARMS) diagnoses and the original 92-item version of the Prodromal Questionnaire (PQ-92) as tested by Ising et al. in a Dutch general help-seeking population with a mean age of 26.2 years [21]. Socioeconomic status of the participant’s family was assessed with the Family Affluence Scale (FAS) [23]. The FAS had a low internal consistency in this study with an ordinal alpha of 0.42. Andersen et al. showed that in six European countries the FAS completed by 11-year-old adolescents had good validity with the parental answers to the FAS questions [24]. Yes/no questions were used to determine whether participants experienced the divorce or death of a parent. Self-esteem was measured with the Rosenberg Self-Esteem scale (RSE), a 10-item questionnaire with both positive and negative statements about self-esteem [25]. Responses are given on a four-point scale (0, totally agree; 1, agree; 2, disagree; 3, totally disagree), with the total score ranging from 0 to 30. The RSE questionnaire had a high internal consistency in this study with an ordinal alpha of 0.87. The RSE as tested in 13–18-year-old adolescents (mean age 15.3 years) from 13 countries by Berry et al. had a Cronbach alpha of 0.83 in nationals and 0.75 in immigrants, which is comparable to the alpha calculated in our study [26]. Screening for psychosocial problems was performed with the Strengths and Difficulties Questionnaire (SDQ) [27, 28]. The SDQ contains 25 items assessing strengths and difficulties in the following domains: hyperactivity, emotional symptoms, conduct problems, peer problems, and prosocial behaviour. Each subscale consists of five items on a three-point scale (0, not true; 1, somewhat true; 2, certainly true). The total score of each subscale therefore ranges from 0 to 10. The internal consistency in this study of the SDQ subscale hyperactivity was high with an ordinal alpha of 0.83. The internal consistency of the SDQ subscales emotional symptoms, conduct problems, prosocial behaviour and peer problems was acceptable with ordinal alpha’s of 0.66, 0.62, 0.67 and 0.50 respectively. The concurrent validity of the SDQ, as tested in a large sample of non-clinical Dutch children and adolescents (*n* = 562; mean age 12.3 years) by Muris et al. [29], was good. Peer status was measured via peer nominations according to the method of Coie et al. [30]. The following five social status groups were defined: popular, rejected, neglected, controversial and average. The categories popular, controversial and average were classified as high peer status, the other two categories as low peer status. The full description of this method can be found in El Bouhaddani et al. [19] and Coie et al. [30]. The adolescents completed the Social And Health Assessment (SAHA) somatic symptoms scale, consisting of 12 items representing commonly reported somatic symptoms by children and adolescents [31]. It assesses the presence of these symptoms during the past month on a three-point scale (0, not true; 1, somewhat true; 2, certainly true). The internal consistency of the SAHA somatic symptoms scale in this study was acceptable with an ordinal alpha of 0.76. This alpha is comparable to the Cronbach alpha of 0.81 that has been previously calculated in the general population of adolescents [32].

### Measurements at follow-up

At 12-month follow-up, the PQ-16 including distress severity was again used to assess whether participants had experienced distressing AVH during the follow-up period. Furthermore, yes/no questions were used to determine whether participants had felt discriminated on skin colour, ethnicity or religion, or had experienced the divorce or death of a parent during the follow-up period. Lifetime presence of trauma was assessed with six items derived from the post-traumatic stress disorder (PTSD) section of the Diagnostic Interview Schedule for Children (DISC) [33]. The interview questions were transformed to self-report yes/no questions and previously used in a study by Adriaanse et al. [34]. Participants were asked to answer questions from the SAHA questionnaire about substance abuse. Having ever used alcohol or cannabis was assessed on a four-point scale (0, never; 1, once; 2, a few times; 3, more than a few times; which was recoded to 0, no; 1, yes), and having been drunk or having been in a fight while drinking alcohol in the past year was determined with a different four-point scale (0, never; 1, 1–2 times; 2, 3–5 times; 3, 6 or more times; which was recoded to 0, no; 1, yes) [31]. An adjusted version of the mastery questionnaire was used to measure the amount of locus of control [35]*.* This questionnaire contains six items on a five-point scale (0, totally disagree; 1, somewhat disagree; 2, intermediate; 3, somewhat agree; 4, totally agree). The internal consistency of the mastery questionnaire in this study was acceptable with an ordinal alpha of 0.73. We do not have further information on the validity of this adjusted version of the mastery questionnaire in adolescents. Finally, participants were asked if they were religious (0, no; 1, somewhat; 2, very; 3, unsure). No and unsure were coded as non-religious, somewhat and very as religious. The variables that were obtained at the follow-up assessment were included in the analyses because these provided additional information about the period before follow-up.

### Data analysis

Potential predictors were selected based on a data-driven approach. Therefore, all available variables were used, except those that showed high inter-correlations (≥ 0.8), were missing for over 25% of cases or were formulated unclearly. Descriptive statistics of the sample were calculated using pooled means, standard deviations, frequency counts and percentages across the imputed datasets. Pearson’s Chi-square tests (*χ*^2^) and non-parametric Mann–Whitney *U* tests (*U*) were used to identify differences between participants included and excluded in this study. Internal consistency of the used questionnaires in this study was estimated with ordinal alpha’s, which were calculated based on the polychoric item correlation matrix [36]. Internal consistency was considered acceptable when alpha > 0.7 [37]. Missing values were imputed ten times with multiple imputation, using the R-package ‘multivariate imputation by chained equations’ (MICE) [38]. Predictors of spontaneous discontinuation of AVH were investigated using least absolute shrinkage and selection operator (LASSO) analyses in each of the imputed datasets [39]. LASSO allows for identification of the most important predictors of a given outcome from a large set of candidate predictors by using regularization, which shrinks unimportant predictors to zero, while maintaining the most important predictors in the model. This procedure has the advantage that it allows for identification of a model that balances model parsimony with prediction accuracy. The number of selected predictors depends on the magnitude of the penalty/shrinkage, for which an optimal value needs to be selected. To do this, the optimal value of the tuning parameter λ was selected via fivefold cross-validation. This entailed selecting the λ value that was associated with the best prediction [highest area under the receiver operating curve (ROC)] across the five folds. The associated model was selected and, to guard against overfitting, the coefficients of the most parsimonious model within 1 standard error (SE) from the selected model were used for model interpretation. LASSO analyses were run using the ‘glmnet’ R-package [40]. The results of the LASSO analyses were tallied across the 10 runs. The predictors that appeared in at least 8 of the 10 imputed datasets were selected. Finally, to get an idea of the joint prediction of the selected predictors in a more conventional statistical approach, they were combined in a multivariable model. Statistical analyses were carried out with R (version 3.5.2; R-core team, 2018) and the Statistical Package for the Social Sciences version 23 for Windows (SPSS 23.0; IBM Inc. New York, USA).

## Results

### Description of the study population

A total of 251 of the 1841 (14.0%; 59.2% female) adolescents reported AVH at baseline. 160 adolescents (8.7% of the total sample; 60.4% female) mentioned having experienced distress from the AVH. Of those 160 adolescents, 124 (77.5%) had completed their follow-up questionnaire. One case was removed, because analyses showed clear response tendency which made the data of this participant unreliable. The remaining 123 participants were included in the analyses. The mean age of the included participants was 12.6 years (range 12–14 years), 62.6% of them were female, 29.6% were migrant-Dutch and 52.0% attended a higher educational level. All sample characteristics are given in Table 1.

**Table 1:** Characteristics of the total group of participants with distressing AVH (*N* = 123) plus split for the persistence (*N* = 43) and discontinuation (*N* = 80) groups

Characteristics	All *N* = 123	Persistence *N* = 43	Discontinuation *N *= 80
Age (years), mean (SD)	12.63 (0.62)	12.56 (0.67)	12.66 (0.59)
Male gender, *N *(%)	46 (37.4)	15 (34.9)	31 (38.8)
Not born in The Netherlands, *N *(%)	4 (3.3)	1 (2.3)	3 (3.8)
Migrant-Dutch ethnicity, *N *(%)	36 (29.6)	14 (33.3)	22 (27.6)
Religious, *N *(%)	43 (35.0)	15 (34.9)	28 (35.0)
Living in a large town (> 110.000 residents), *N *(%)	92 (74.8)	32 (74.4)	60 (75.0)
Number of children in the family (including participant), mean (SD)	2.58 (1.34)	2.60 (1.50)	2.56 (1.26)
High socioeconomic status of the family, *N *(%)	92 (74.8)	29 (67.4)	63 (78.8)
High education level, *N *(%)	64 (52.0)	25 (58.1)	39 (48.8)
School grade repetition, *N *(%)	32 (26.0)	6 (14.0)	26 (32.5)
Moved houses (ever), *N *(%)	73 (59.6)	26 (60.7)	47 (59.0)
Parents divorced, *N* (%)	31 (25.1)	11 (25.4)	20 (25.0)
Parents divorced in past year, *N *(%)	8 (6.5)	6 (14.0)	2 (2.5)
Parent deceased, *N *(%)	3 (2.4)	1 (2.3)	2 (2.5)
Parent deceased in past year, *N *(%)	3 (2.4)	2 (4.7)	1 (1.3)
Traumatic experiences: *N* (%)			
Been scared that an acquaintance would be killed or seriously injured	56 (45.5)	24 (55.8)	32 (40.0)
Attacked, beaten up or threatened	47 (38.2)	18 (41.9)	29 (36.3)
Sexually assaulted	10 (8.1)	6 (14.0)	4 (5.0)
Been in or near a serious accident	40 (32.5)	14 (32.6)	26 (32.5)
Seen or heard someone being killed, passed away or seriously injured	37 (30.1)	14 (32.6)	23 (28.8)
Been scared by seeing a deceased body	40 (32.4)	18 (41.9)	22 (27.3)
Been discriminated on skin colour, ethnicity or religion in past year, *N* (%)	13 (10.6)	4 (9.3)	9 (11.3)
Substance use: *N *(%)			
Alcohol (ever)	54 (43.9)	23 (53.5)	31 (38.8)
Been drunk in past year	9 (7.6)	6 (14.0)	3 (4.3)
Been in a fight while drinking alcohol in past year	4 (3.3)	2 (4.7)	2 (2.6)
Cannabis (ever)	9 (7.0)	6 (14.0)	3 (3.3)
SAHA somatic complaints (range 0–20), mean (SD)	8.32 (5.00)	8.85 (5.29)	8.03 (4.85)
SDQ scales: mean (SD)			
Hyperactivity (range 0–10)	5.03 (2.58)	4.63 (2.96)	5.25 (2.34)
Prosocial behaviour (range 0–10)	7.90 (1.59)	8.21 (1.74)	7.73 (1.48)
Conduct problems (range 0–10)	2.51 (1.65)	2.28 (1.55)	2.64 (1.70)
Emotional problems (range 0–10)	4.11 (2.30)	4.07 (2.20)	4.14 (2.36)
Peer problems (range 0–10)	2.55 (1.78)	2.40 (1.81)	2.64 (1.77)
RSE (self-esteem) total score (range 0–30), mean (SD)	16.49 (2.99)	16.15 (2.41)	16.68 (3.26)
Mastery total score (range 0–26), mean (SD)	17.63 (3.61)	17.72 (3.85)	17.57 (3.50)
High peer status, *N *(%)	60 (48.8)	22 (51.2)	38 (47.5)
PQ-16 psychotic experiences: *N *(%)			
I feel uninterested in the things I used to enjoy	24 (19.5)	9 (20.9)	15 (18.8)
I often live through events exactly as they happened before	62 (50.4)	16 (37.2)	46 (57.5)
I sometimes smell or taste things that other people do not notice	21 (16.8)	7 (15.8)	14 (17.3)
I often hear unusual sounds in my ears	79 (64.1)	26 (60.7)	53 (65.9)
I have been confused at times whether something I experienced was real or imaginary	43 (35.0)	15 (34.9)	28 (35.0)
When I look at a person or at myself in a mirror, I have seen the face change right before me	21 (17.2)	5 (11.6)	16 (20.1)
I get extremely anxious when meeting people for the first time	37 (30.1)	9 (20.9)	28 (35.0)
I have seen things that other people cannot or do not see	51 (41.5)	20 (46.5)	31 (38.8)
My thoughts are sometimes so strong that I can almost hear them	38 (30.9)	14 (32.6)	24 (30.0)
I sometimes see special meanings in advertisements, shop windows, or in the way things are arranged around me	14 (11.4)	4 (9.3)	10 (12.5)
I have felt not in control of my own ideas or thoughts	47 (38.2)	16 (37.2)	31 (38.8)
I sometimes feel suddenly distracted by distant sounds that I am not normally aware of	75 (61.1)	27 (63.3)	48 (60.0)
I often feel that others have it in for me	37 (30.2)	7 (16.3)	30 (37.8)
I have had the sense that a person or force is around me, even though I did not see anyone	44 (35.9)	18 (41.9)	26 (32.6)
I feel that parts of my body have changed in some way or that parts of my body are working differently	16 (13.3)	2 (4.7)	14 (17.9)

*AVH* auditory verbal hallucinations, *N* number of participants, *PQ-16* 16 item prodromal questionnaire, *RSE* Rosenberg self-esteem scale, *SAHA* social and health assessment, *SD* standard deviation, *SDQ* strengths and difficulties questionnaire (for age 4–17 years)

### Non-response

The percentage of non-response from all invited adolescents at the baseline screening was 14.4% (310/2151 adolescents). 8.8% of these adolescents had no consent, 4.9% was not present during the baseline screening because of illness or unknown reason, and 0.7% of the adolescents had changed schools. The percentage of non-response at the follow-up screening was 15.3% (329/1841 adolescents). 1.0% of these adolescents had no consent, 8.2% was not present during the follow-up screening because of illness or unknown reason, 0.9% had changed schools, and in 7.8% the school had stopped its participation in the study.

### Dropout analysis

23% (*n* = 36) of the adolescents who experienced distress from AVH at baseline did not participate in the follow-up measurement. The dropouts differed from the subjects included in this study on several characteristics: dropouts more often had divorced parents (55.6% of dropouts vs. 24.6% of participants, *χ*^2^ = 12.3, *p* = 0.000), were more frequently born abroad (16.7% vs. 3.3%, *χ*^2^ = 8.5, *p* = 0.004) and had more conduct problems (3.39 vs. 2.51, *U* = 1645, *p* = 0.017).

### Discontinuation rates

**Fig. 2:**
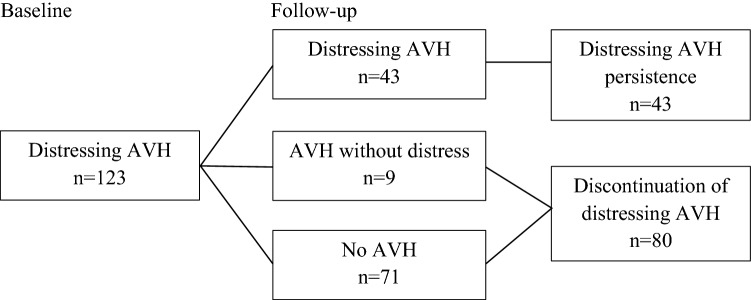
Flowchart outlining the follow-up groups

During follow-up, 43 adolescents (35.0%) reported that they had experienced at least mildly distressing AVH during the last 12 months. In that period, nine adolescents (7.3%) had experienced AVH without distress and 71 adolescents (57.7%) had not experienced AVH. Thus, in 80 adolescents (65.0%) the distressing AVH had spontaneously discontinued. The flowchart outlining the follow-up groups is depicted in Fig. 2.

### Predictors of spontaneous discontinuation at follow-up

The results of the LASSO analyses are presented in Table 2. The overall predictive value of the models ranged between AUC = 0.58 and AUC = 0.62 across the imputed datasets (i.e. 0.5 indicates chance prediction, 1.0 indicates perfect prediction), indicating that some predictive value, although limited, was present. The results also indicated some potentially important predictors of spontaneous discontinuation of distressing AVH at 12-month follow-up. Nine predictors were consistently selected across the imputed datasets (in  ≥ 8 imputed datasets). These were: never having used cannabis, parents not being divorced in the past year, never having been scared by seeing a deceased body, less prosocial behaviour, having repeated a school grade, having the feeling that others have it in for you, having anxiety when meeting new people, having lived through events exactly as if they happened before and having the feeling as if parts of the body have changed.

**Table 2:** Results of LASSO-penalized logistic regression with spontaneous discontinuation of distressing AVH at 12 months after baseline as outcome

	Imputed datasets									
	1	2	3	4	5	6	7	8	9	10
(Intercept)	2.74	0.60	1.00	0.97	2.15	0.69	1.95	3.53	2.77	1.10
Age	–	–	–	–	–	–	–	− 0.13	–	–
Male gender	–	–	–	–	–	–	–	–	–	–
Not born in The Netherlands	0.68	–	-–	–	0.62	–	0.39	1.34	0.80	–
Migrant-Dutch ethnicity	–	–	–	–	–	–	–	–	–	–
Religious	–	–	–	–	–	–	–	0.09	–	–
Living in a large town ( > 110,000 residents)	–	–	–	–	–	–	–	–	–	–
Number of children in the family (including participant)	− 0.12	–	–	–	− 0.12	–	– 0.06	− 0.23	– 0.14	–
High socioeconomic status of the family	0.36	–	–	–	0.34	–	0.24	0.54	0.37	0.11
High education level	–	–	–	-–	–	–	–	–	–	–
**School grade repetition**	**1.19**	–	**0.37**	**0.38**	**1.16**	–	**0.96**	**1.66**	**1.24**	**0.62**
Moved houses (ever)	–	–	–	–	–	–	–	− 0.16	–	–
Parents divorced	0.47	–	–	–	0.44	–	0.20	1.06	0.58	–
**Parents divorced in past year**	**− 1.73**	**− 0.11**	**− 0.86**	**− 0.90**	**− 1.75**	**− 0.19**	**− 1.38**	**− 2.01**	**− 1.72**	**− 1.16**
Parent deceased in past year	− 0.64	–	–	–	− 0.31	–	–	− 0.32	− 1.08	− 0.69
Traumatic experiences										
Been scared that an acquaintance would be killed or seriously injured	–	–	–	–	–	–	–	–	–	− 0.01
Attacked, beaten up or threatened	–	–	–	–	–	–	–	–	–	–
Sexually assaulted	–	–	–	− 0.04	− 0.01	–	− 0.07	–	–	− 0.12
Been in or near a serious accident	–	–	–	–	–	–	–	–	–	–
Seen or heard someone being killed, passed away or seriously injured	–	–	–	–	–	-	–	0.07	–	–
**Been scared by seeing a deceased body**	**– 0.63**	**–**	**− 0.05**	**-0.05**	**− 0.62**	**–**	**− 0.47**	**− 0.93**	**− 0.71**	**− 0.30**
Been discriminated on skin colour, ethnicity or religion in past year	–	–	–	–	–	–	–	–	–	–
Substance use										
Alcohol (ever)	− 0.22	–	–	–	− 0.22	–	− 0.15	− 0.40	− 0.27	–
Been drunk in past year	–	–	–	–	–	–	–	–	–	–
Been in a fight while drinking alcohol in past year	− 0.42	–	− 0.11	–	– 0.51	–	− 0.06	− 0.66	− 0.46	–
**Cannabis (ever)**	**− 1.56**	**–**	**− 0.15**	**− 0.18**	**− 1.24**	**–**	**− 1.32**	**− 2.34**	**– 1.71**	**− 0.53**
SAHA somatic complaints	–	–	–	–	–	–	− 0.01	–	− 0.01	–
SDQ scales										
Hyperactivity	0.10	–	–	–	0.10	–	0.07	0.14	0.11	0.03
**Prosocial behaviour**	**– 0.26**	–	**− 0.08**	**− 0.07**	**− 0.25**	–	**− 0.20**	**− 0.34**	**− 0.27**	**− 0.12**
Conduct problems	–	–	–	–	–	–	–	–	–	–
Emotional problems	–	–	–	–	–	–	–	–	–	–
Peer problems	–	–	–	–	–	–	–	–	–	–
RSE (self-esteem) total score	–	–	–	–	–	–	–	–	–	–
Mastery total score	–	–	–	–	–	–	–	–	–	–
High peer status	–	–	–	–	–	–	–	–	–	–
PQ-16 psychotic experiences										
I feel uninterested in the things I used to enjoy	− 0.34	–	–	–	− 0.30	–	− 0.15	− 0.66	− 0.38	
**I often live through events exactly as they happened before**	**0.64**	**–**	**0.22**	**0.24**	**0.64**	**–**	**0.55**	**0.74**	**0.64**	**0.33**
I sometimes smell or taste things that other people do not notice	–	–	–	–	–	–	–	0.17	–	–
I often hear unusual sounds in my ears	–	–	–	–	–	–	–	–	–	–
I have been confused at times whether something I experienced was real or imaginary	–	–	–	–	–	–	–	–	–	–
When I look at a person or at myself in a mirror, I have seen the face change right before me	0.24	–	–	–	0.27	–	0.08	0.33	0.24	–
**I get extremely anxious when meeting people for the first time**	**0.35**	**–**	**0.01**	**0.02**	**0.34**	**–**	**0.24**	**0.39**	**0.32**	**0.10**
I have seen things that other people cannot or do not see	–	–	–	–	–	–	–	–	–	–
My thoughts are sometimes so strong that I can almost hear them	− 0.01	–	–	–	− 0.02	–	–	− 0.12	− 0.02	–
I sometimes see special meanings in advertisements, shop windows, or in the way things are arranged around me	–	–	–	–	–	–	–	–	–	–
I have felt not in control of my own ideas or thoughts	−	–	–	–	–	–	–	–	–	–
I sometimes feel suddenly distracted by distant sounds that I am not normally aware of	– 0.33	–	–	–	− 0.33	–	− 0.01	− 0.35	− 0.18	−
**I often feel that others have it in for me**	**–**	**0.10**	**0.43**	**0.43**	**1.06**	**0.10**	**0.69**	**1.39**	**1.07**	**0.62**
I have had the sense that a person or force is around me, even though I did not see anyone	–	–	–	–	− 0.02	–	0.00	− 0.10	− 0.05	–
**I feel that parts of my body have changed in some way or that parts of my body are working differently**	**0.11**	**–**	**0.11**	**0.05**	**0.24**	**–**	**0.19**	**0.26**	**0.18**	**0.25**
AUC of best model	0.64	0.62	0.61	0.62	0.64	0.61	0.62	0.64	0.62	0.62
SE (AUC of best model)	0.03	0.04	0.04	0.04	0.03	0.03	0.02	0.03	0.02	0.03
AUC of selected most parsimonious model	0.61	0.62	0.58	0.58	0.61	0.60	0.60	0.62	0.60	0.60

Presented coefficients are for the most parsimonious solution in each dataset. Coefficients are provided for the lambda value of the most parsimonious model with an AUC within 1 SE from the optimal cross-validated model. Note: coefficients are beta-coefficients for the prediction of the log-odds of the outcome. ORs can be obtained by taking the exponent of the beta coefficients. All coefficients regularized to zero are replaced by ‘–‘ for clarity. All variables with a non-zero coefficient in ≥ 8 of the 10 imputed datasets are printed in bold

*AUC *area under the curve, *AVH* auditory verbal hallucinations, *OR* odds ratio, *PQ-16* 16 item prodromal questionnaire, *RSE* Rosenberg self-esteem scale, *SAHA* social and health assessment, *SDQ* strengths and difficulties questionnaire (for age 4–17 years), *SE* standard error

In Table 3, the results of a conventional multivariable regression model with only the selected predictors is shown. The results of these analyses indicated that only four of the nine selected predictors showed a significant multivariate association, although all nine were found to contribute substantially to the prediction of AVH persistence in the LASSO analyses.

**Table 3:** Pooled results from the multivariable logistic regression analyses of the predictors of spontaneous discontinuation of distressing AVH at 12 months after baseline (*N* = 123)

Predictors	OR	95% CI low	95% CI high
(Intercept)	26.32	1.94	357.09
School grade repetition	5.09	1.47	17.64*
SDQ prosocial behaviour	0.66	0.48	0.90*
PQ-16: ‘I often live through events exactly as they happened before’	2.40	0.91	6.29
PQ-16: ‘I get extremely anxious when meeting people for the first time’	2.01	0.67	6.07
PQ-16: ‘I often feel that others have it in for me’	3.56	1.08	11.68*
PQ-16: ‘I feel that parts of my body have changed in some way or that parts of my body are working differently’	1.59	0.28	9.06
Been scared by seeing a deceased body	0.46	0.18	1.16
Cannabis (ever)	0.19	0.03	1.45
Parents divorced in past year	0.07	0.01	0.59*

*AVH *auditory verbal hallucinations, *CI* confidence interval, *OR* odds ratio, *N* number of participants, *PQ-16* 16 item prodromal questionnaire, *RSE* Rosenberg self-esteem scale, *SAHA* social and health assessment, *SDQ* strengths and difficulties questionnaire (for age 4–17 years)

*p < 0.05

Post hoc analysis of the prosocial behaviour data showed that adolescents with the lowest scores (scores 3–4/10) and the highest scores (scores 9–10/10) on the SDQ prosocial behaviour scale were part of the distressing AVH persistence group, while the adolescents with intermediate scores (scores 5–8/10) were part of the discontinuation group.

## Discussion

In the school-based sample of 1841 adolescents, 14% had experienced AVH with or without distress. 64% of the adolescents with AVH reported at baseline that they had experienced AVH that distressed them. The majority (65%) of the adolescents who participated in the follow-up did not experience distressing AVH during the follow-up period. Discontinuation of distressing AVH could not be predicted by socio-demographic characteristics, but was predicted by never having used cannabis, parents not being divorced in the past year, never having been scared by seeing a deceased body, less prosocial behaviour, having repeated a school grade, having the feeling that others have it in for you, having anxiety when meeting new people, having lived through events exactly as if they happened before and having the feeling as if parts of the body have changed.

The prevalence rate of AVH in our sample is similar to rates presented in the meta-analysis by Kelleher et al. [1]. It should be noted that distress was often not taken into account in the studies included in that meta-analysis. The discontinuation rate of distressing AVH is lower than the 2-year AVH discontinuation rate of 73% reported in a similar population-based study among 13–14-year-old adolescents by De Loore et al. [11]. Possibly, if we would have assessed the participants again 2 years after baseline, more participants might have discontinued from distressing AVH and the discontinuation rate might have been closer to or even higher than the rate reported by De Loore et al. [11].

Inherent to the data-driven approach is that, next to finding theoretically expected factors associated with discontinuation of distressing AVH, factors were found that are theoretically difficult to interpret and/or cannot be influenced in the daily life of adolescents with distressing AVH. Although the predictors were selected based on their joined ability to predict the outcome, the individual predictors are further elaborated on in the next paragraphs, to outline potential theoretical explanations for their contribution to the model.

In this study, younger age and not being from an ethnic minority group did not predict discontinuation of distressing AVH. However, never having used cannabis, parents not being divorced in the past year and never having been scared by seeing a deceased body were found to predict spontaneous discontinuation from distressing AVH. This is in line with the findings of several studies [6, 12, 13]. In The Netherlands, 1.5–6.8% of 13–14-year-old adolescents have ever used cannabis [41]. In the current study, 2.6% of the adolescents with discontinuation of distressing AVH reported ever having used cannabis, while this was much higher (14.0%) in the persistence group. We hypothesized that the divorce of parents and being scared by seeing a deceased body represent highly emotional and, for some adolescents, traumatic events. Not having experienced such traumatic events can therefore be regarded as a protective factor for persistence of distressing AVH. Substance abuse and trauma are not only associated with AVH persistence, but they are also well-known environmental risk factors for the development of psychosis and other mental illness [42, 43].

Less prosocial behaviour as a predictor of spontaneous discontinuation from distressing AVH might seem counterintuitive, because prosocial behaviour in adolescence has been associated with a variety of positive outcomes such as high self-esteem, academic success and high-quality relationships [44]. When looking more closely to the data to explore this finding, we found that adolescents with the lowest and highest scores on the SDQ prosocial behaviour scale were part of the distressing AVH persistence group. This is in line with literature stating that childhood psychopathology is associated with both high and low levels of prosocial behaviour [45–47]. It can be argued that both low and high prosocial behaviour indicate problems with social interactions, which in turn increase the risk of psychiatric problems. In those social interactions, adolescents with low prosocial behaviour tend to lack empathy towards others. Excessive prosocial behaviour, however, might reflect sub-assertiveness; a strong tendency to please others. We do not know the direction of the association between abnormal prosocial behaviour and persistence of distressing AVH. Therefore, more research on the relationship between prosocial behaviour and discontinuation of distressing AVH in other non-clinical adolescent cohorts is needed.

A novel finding is that the distressing AVH were more likely to discontinue in participants who repeated a school grade. The 95% confidence interval was wide (1.47–17.64). Therefore, this finding must be read with some caution. We did not find an association between the specific year that was repeated and discontinuation from distressing AVH. We do not have a ready explanation for the finding that in our sample school grade repetition was associated with distressing AVH discontinuation. Grade repetition is often considered a consequence of cognitive or social–emotional problems. There is, however, limited knowledge on the effect of grade repetition on the social–emotional development of students [48]. One study reported that 13–14-year-old students who had ever repeated a year had more academic, emotional, and behavioural problems than the non-retained students [49]. There are, to our knowledge, no studies that describe a positive social–emotional effect of grade repetition in a population similar to the current study’s population.

Another novel finding is that having the feeling that others have it in for you was associated with discontinuation of distressing AVH. The feeling that others have it in for you can be regarded as distrust or suspiciousness. Delusional ideation has been associated with persistence of AVH in adolescence [6]. The finding of this study that having the feeling that others have it in for you was associated with discontinuation of distressing AVH was therefore surprising. Other surprising findings from this study were that anxiety when meeting new people, having lived through events exactly as if they happened before and having the feeling as if parts of the body have changed predicted discontinuation of distressing AVH at 12-month follow-up. At this point, we do not have an evident theoretical explanation for these findings. The 95% confidence intervals for these predictors were wide and therefore must be read with caution. Usually, these variables are related to persistence of psychotic experiences and not discontinuation. This raises the question about the validity of the concept AVH in adolescents. Studies on APS found that they are more prevalent in populations under the age of 15/16 years than in older adolescents. At the same time, APS less frequently cause functional impairment or mental disorders in the younger age category, indicating that AVH have less clinical significance in younger adolescents, even when they experience distress from the AVH [5, 18, 50–52]. Potentially, even when young adolescents experience distress from the AVH, they are less related to psychopathology than we hypothesized.

### Strengths and limitations

This study has some limitations. First, voice hearing was assessed with one question: “I have heard things other people cannot hear, like voices of people whispering or talking”. At baseline, this question was not specified by a time period, such as ‘in the past month’. This could have led to differences in the way the question was interpreted. If participants interpreted the question as ‘have you *ever* in your life heard things other people cannot hear, like voices of people whispering or talking’, we could have included participants in whom the distressing AVH had already discontinued. Nevertheless, the prevalence rates of (distressing) AVH at baseline were conform prevalence rates of AVH in adolescents (ranging from 4.7 to 35.3%) as presented in the meta-analysis of Kelleher et al. [1]. Furthermore, the number, type and hostility of the voices and the degree of reality attached to the distressing AVH were not investigated. Therefore, the assessment of the distressing AVH was limited. Another limitation is that we were not able to research all predictors of AVH persistence found in previous studies (e.g. age of onset of AVH and developmental problems), since these were not assessed in the MasterMind study [19]. Next, follow-up data was only obtained once at 12 months after baseline and participants were not asked whether they received mental health care. It therefore remains unknown from this study whether distress associated with the AVH predicts a need for mental health care. A potential limitation is that this study relied on the PQ-16, a self-report questionnaire, to measure AVH [21]. Although the percentage of AVH in adolescents found in this study is in line with rates reported in previous non-clinical adolescent samples, data might have been prone to response bias. Participants might have misunderstood the questions or might have responded to questions in a ‘socially desirable way’, even though the questionnaires were anonymous [53]. A recent review of screening instruments for identifying individuals at clinical high risk for psychosis concluded that the majority of measures, including the PQ-16, have relatively poor or underexplored psychometric properties [54]. Although the current study did not have the objective to identify adolescents at clinical high risk for psychosis, the PQ-16 might not have been the most optimal method to measure AVH, risking overestimation of AVH. However, a previous study by Kelleher et al. in 11–13-year-old non-clinical adolescents found that self-reported AVH, defined as a positive answer to the question “Have you ever heard voices or sounds that no one else can hear?”, had good positive predictive value (71.4%) and good negative predictive value (90.4%) when compared to AVH assessed via a clinical interview [1]. In the current study we used a positive answer to the statement “I have heard things other people cannot hear, like voices of people whispering or talking” to determine the presence of AVH, which is a similar question to the one asked by Kelleher et al. To increase clinical significance and to reduce overestimation of AVH prevalence, we rated a positive answer on this item only as AVH if adolescents reported distress associated with the experience. Also a limitation is that one of the questionnaires, the FAS, had a low internal consistency in this study. This indicates that in this study the items of the FAS did not fully measure the same construct or that the answers to the items were inconsistent. Because the FAS did not predict the outcome in 8 or more of the imputed datasets and it therefore was not regarded as a predictor of spontaneous discontinuation of distressing AVH, we did not take additional steps to remove it from analyses. Next, it might have added extra value to the study if a linear multivariable regression analysis with ‘no AVH’, ‘AVH without distress’ and ‘distressing AVH’ as outcome groups would have been performed, because this might have differentiated the risk of future mental health problems. However, since only nine participants reported AVH without distress, such analysis was not possible. Finally, almost a quarter (22.5%) of the participants with distressing AVH at baseline did not participate in the follow-up. Reasons for dropout at follow-up were: not present at follow-up measurement, participants or their parents withdrew their consent or school withdrew their consent. Analysis of the dropout population revealed that dropouts more often had divorced parents, were more frequently born abroad and had more conduct problems. These factors are potentially related to a higher risk of distressing AVH persistence. This implies that the included participants comprised a group with more favourable outcome and therefore discontinuation rates might be overestimated.

The main strengths of this study are that it is based on a large school-based sample of young adolescents and that it includes a large number of variables. Because this is a population-based sample, adolescents of all socio-demographic backgrounds and educational levels are included. Furthermore, this is one of few AVH studies investigating adolescents instead of young children or adults. Since adolescence is the period during which the first signs of psychopathology often emerge, more research specifically on adolescents with AVH is needed. Another strength of the current study is that the study’s description of AVH took distress into account. Persistence of distressing AVH in healthy individuals increases the risk of mental health problems [14]. Therefore, using distress of AVH as an extra outcome criterion helps differentiating between adolescents at risk and not at risk of mental health problems. This increases clinical significance of the current study. Finally, we have used the LASSO method for our statistical analyses. This technique allowed us to identify the most important predictors for spontaneous discontinuation of distressing AVH within the large set of potential predictors. The difference in significant predictors found by the LASSO analyses and the conventional multivariable regression analysis indicates that important predictors can be missed if selected only based on conventional forward regression strategies.

## Conclusion

The rates presented in the current study suggest that distressing AVH in healthy adolescents are common, but transitory in approximately two-thirds of those adolescents. This study’s results show that it is possible to predict discontinuation of AVH in this population up to a certain extent. However, several predictors were difficult to interpret and, except for cannabis use, do not provide leads for screening or preventive measures at schools.

## Data availability

The dataset used and analysed during the current study is available from the corresponding author on reasonable request.

## Electronic supplementary material

Below is the link to the electronic supplementary material.

Supplementary file1 (DOCX 103 kb)

## Abbreviations

AUC: Area under the curve
APS: Attenuated psychotic symptoms
AVH: Auditory verbal hallucinations
CAARMS: Comprehensive assessment of at-risk mental states
CI: Confidence interval
DISC: Diagnostic interview schedule for children
FAS: Family affluence scale
LASSO: Least absolute shrinkage and selection operator
MICE: Multivariate imputation by chained equations
*N*: Number of participants
OR: Odds ratio
PE: Psychotic experiences
PQ-16: 16-Item prodromal questionnaire
PQ-92: 92-Item prodromal questionnaire
PTSD: Post-traumatic stress disorder
ROC: Receiver operating curve
RSE: Rosenberg self-esteem scale
SAHA: Social and health assessment scale
SD: Standard deviation
SDQ: Strengths and difficulties questionnaire (for age 4–17 years)
SE: Standard error
SPSS: Statistical package for the social sciences
*U*: Non-parametric Mann–Whitney *U* tests
χ^2^: Pearson’s Chi-square test
