# Supplementary material for: Spontaneous discontinuation of distressing auditory verbal hallucinations in a school-based sample of adolescents: a longitudinal study
Source: Eur Child Adolesc Psychiatry. 2019 Aug 27;29(6):777–90. doi: 10.1007/s00787-019-01393-7 (PMC7305260; doi:10.1007/s00787-019-01393-7)
Supplement: Supplementary file 1 — Supplementary file1 (DOCX 103 kb) [file 787_2019_1393_MOESM1_ESM.docx]

***Supplement 1***

Characteristics of the total group of participants with distressing AVH (N=123) in all sets.

| Characteristics | 1 | 2 | 3 | 4 | 5 | 6 | 7 | 8 | 9 | 10 |
| --- | --- | --- | --- | --- | --- | --- | --- | --- | --- | --- |
| Age (years) *mean (SD)*  Male gender *N (%)*  Not born in The Netherlands *N (%)*  Migrant-Dutch ethnicity *N (%)*  Religious *N (%)*  Living in a large town (>110.000 residents) *N (%)*  Number of children in the family (including participant) *mean (SD)*  High socioeconomic status of the family *N (%)*  High education level *N (%)*  School grade repetition *N (%)*  Moved houses (ever) *N (%)*  Parents divorced *N (%)*  Parents divorced in past year *N (%)*  Parent deceased *N (%)*  Parent deceased in past year *N (%)*  Traumatic experiences: *N (%)*  - Been scared that an acquaintance would be killed or seriously injured  - Attacked, beaten up or threatened  - Sexually assaulted  - Been in or near a serious accident  - Seen or heard someone being killed, passed away or seriously injured  - Been scared by seeing a deceased body  Been discriminated on skin colour, ethnicity or religion in past year *N (%)*  Substance use: *N (%)*  - alcohol (ever)  - been drunk in past year  - been in a fight while drinking alcohol in past year  - cannabis (ever)  SAHA somatic complaints (range: 0-20) *mean (SD)*  SDQ scales: *mean (SD)*  - Hyperactivity (range: 0-10)  - Prosocial behaviour (range: 0-10)  - Conduct problems (range: 0-10)  - Emotional problems (range: 0-10)  - Peer problems (range: 0-10)  RSE (self-esteem) total score (range: 0-30) *mean (SD)*  Mastery total score (range: 0-26) *mean (SD)*  High peer status *N (%)*  PQ-16 psychotic experiences: *N (%)*  - I feel uninterested in the things I used to enjoy.  - I often live through events exactly as they happened before.  - I sometimes smell or taste things that other people do not notice.  - I often hear unusual sounds in my ears.  - I have been confused at times whether something I experienced was real or imaginary.  - When I look at a person, or at myself in a mirror, I have seen the face change right before me.  - I get extremely anxious when meeting people for the first time.  - I have seen things that other people cannot or do not see.  - My thoughts are sometimes so strong that I can almost hear them.  - I sometimes see special meanings in advertisements, shop windows, or in the way things are arranged around me.  - I have felt not in control of my own ideas or thoughts.  - I sometimes feel suddenly distracted by distant sounds that I am not normally aware of.  - I often feel that others have it in for me.  - I have had the sense that a person or force is around me, even though I did not see anyone.  - I feel that parts of my body have changed in some way, or that parts of my body are working differently. | 12.63 (0.62)  46 (37.4)  4 (3.3)  36 (29.3)  43 (35.0)  92 (74.8)  2.58 (1.34)  92 (74.8)  64 (52.0)  32 (26.0)  74 (60.2)  31 (25.2)  8 (6.5)  3 (2.4)  3 (2.4)  56 (45.5)  47 (38.2)  10 (8.1)  40 (32.5)  37 (30.1)  40 (32.5)  13 (10.6)  54 (43.9)  9 (7.3)  4 (3.3)  8 (6.5)    8.29 (4.97)  5.03 (2.58)  7.88 (1.60)  2.51 (1.65)  4.11 (2.30)  2.55 (1.78)  16.53 (2.96)  17.63 (3.61)  60 (48.8)  24 (19.5)  62 (50.4)  22 (17.9)  78 (63.4)  43 (35.0)  21 (17.1)  37 (30.1)  51 (41.5)  38 (30.9)  14 (11.4)  47 (38.2)  76 (61.8)  37 (30.1)  45 (36.6)  16 (13.0) | 12.63 (0.62)  46 (37.4)  4 (3.3)  38 (30.9)  43 (35.0)  92 (74.8)  2.58 (1.34)  92 (74.8)  64 (52.0)  32 (26.0)  74 (60.2)  31 (25.2)  8 (6.5)  3 (2.4)  3 (2.4)  56 (45.5)  47 (38.2)  10 (8.1)  40 (32.5)  37 (30.1)  40 (32.5)  13 (10.6)  54 (43.9)  9 (7.3)  4 (3.3)  9 (7.3)    8.18 (4.96)  5.03 (2.58)  7.92 (1.59)  2.51 (1.65)  4.11 (2.30)  2.55 (1.78)  16.50 (2.99)  17.63 (3.61)  60 (48.8)  24 (19.5)  62 (50.4)  21 (17.1)  79 (64.2)  43 (35.0)  21 (17.1)  37 (30.1)  51 (41.5)  38 (30.9)  14 (11.4)  47 (38.2)  75 (61.0)  38 (30.9)  44 (35.8)  16 (13.0) | 12.63 (0.62)  46 (37.4)  4 (3.3)  37 (30.1)  43 (35.0)  92 (74.8)  2.58 (1.34)  92 (74.8)  64 (52.0)  32 (26.0)  73 (59.4)  31 (25.2)  8 (6.5)  3 (2.4)  3 (2.4)  56 (45.5)  47 (38.2)  10 (8.1)  40 (32.5)  37 (30.1)  40 (32.5)  13 (10.6)  54 (43.9)  9 (7.3)  4 (3.3)  9 (7.3)    8.45 (5.05)  5.03 (2.58)  7.89 (1.59)  2.51 (1.65)  4.11 (2.30)  2.55 (1.78)  16.50 (2.96)  17.63 (3.61)  60 (48.8)  24 (19.5)  62 (50.4)  20 (16.3)  80 (65.0)  43 (35.0)  21 (17.1)  37 (30.1)  51 (41.5)  38 (30.9)  14 (11.4)  47 (38.2)  75 (61.0)  37 (30.1)  44 (35.8)  17 (13.8) | 12.63 (0.62)  46 (37.4)  4 (3.3)  36 (29.3)  43 (35.0)  92 (74.8)  2.58 (1.34)  92 (74.8)  64 (52.0)  32 (26.0)  74 (60.2)  30 (24.4)  8 (6.5)  3 (2.4)  3 (2.4)  56 (45.5)  47 (38.2)  10 (8.1)  40 (32.5)  37 (30.1)  40 (32.5)  13 (10.6)  54 (43.9)  10 (8.1)  4 (3.3)  9 (7.3)    8.24 (4.92)  5.03 (2.58)  7.89 (1.58)  2.51 (1.65)  4.11 (2.30)  2.55 (1.78)  16.52 (2.95)  17.63 (3.61)  60 (48.8)  24 (19.5)  62 (50.4)  18 (14.6)  79 (64.2)  43 (35.0)  22 (17.9)  37 (30.1)  51 (41.5)  38 (30.9)  14 (11.4)  47 (38.2)  75 (61.0)  37 (30.1)  44 (35.8)  16 (13.0) | 12.63 (0.62)  46 (37.4)  4 (3.3)  36 (29.3)  43 (35.0)  92 (74.8)  2.58 (1.34)  92 (74.8)  64 (52.0)  32 (26.0)  74 (60.2)  31 (25.2)  8 (6.5)  3 (2.4)  3 (2.4)  56 (45.5)  47 (38.2)  10 (8.1)  40 (32.5)  37 (30.1)  40 (32.5)  13 (10.6)  54 (43.9)  9 (7.3)  4 (3.3)  9 (7.3)    8.41 (5.10)  5.03 (2.58)  7.90 (1.58)  2.51 (1.65)  4.11 (2.30)  2.55 (1.78)  16.49 (2.97)  17.63 (3.61)  60 (48.8)  24 (19.5)  62 (50.4)  19 (15.5)  79 (64.2)  43 (35.0)  21 (17.1)  37 (30.1)  51 (41.5)  38 (30.9)  14 (11.4)  47 (38.2)  76 (61.8)  37 (30.1)  44 (35.8)  16 (13.0) | 12.63 (0.62)  46 (37.4)  4 (3.3)  36 (29.3)  43 (35.0)  92 (74.8)  2.58 (1.34)  92 (74.8)  64 (52.0)  32 (26.0)  72 (58.5)  31 (25.2)  8 (6.5)  3 (2.4)  3 (2.4)  56 (45.5)  47 (38.2)  10 (8.1)  40 (32.5)  37 (30.1)  39 (31.7)  13 (10.6)  54 (43.9)  10 (8.1)  4 (3.3)  9 (7.3)    8.43 (5.03)  5.03 (2.58)  7.90 (1.58)  2.51 (1.65)  4.11 (2.30)  2.55 (1.78)  16.54 (3.00)  17.63 (3.61)  60 (48.8)  24 (19.5)  62 (50.4)  21 (17.1)  79 (64.2)  43 (35.0)  21 (17.1)  37 (30.1)  51 (41.5)  38 (30.9)  14 (11.4)  47 (38.2)  75 (61.0)  37 (30.1)  44 (35.8)  17 (13.8) | 12.63 (0.62)  46 (37.4)  4 (3.3)  36 (29.3)  43 (35.0)  92 (74.8)  2.58 (1.34)  92 (74.8)  64 (52.0)  32 (26.0)  72 (58.5)  31 (25.2)  8 (6.5)  3 (2.4)  3 (2.4)  56 (45.5)  47 (38.2)  10 (8.1)  40 (32.5)  37 (30.1)  40 (32.5)  13 (10.6)  54 (43.9)  10 (8.1)  5 (4.1)  8 (6.5)    8.25(4.92)  5.03 (2.58)  7.90 (1.58)  2.51 (1.65)  4.11 (2.30)  2.55 (1.78)  16.41 (3.08)  17.63 (3.61)  60 (48.8)  24 (19.5)  62 (50.4)  21 (17.1)  79 (64.2)  43 (35.0)  22 (17.1)  37 (30.1)  51 (41.5)  38 (30.9)  14 (11.4)  47 (38.2)  75 (61.0)  36 (29.3)  44 (35.8)  16 (13.0) | 12.63 (0.62)  46 (37.4)  4 (3.3)  36 (29.3)  43 (35.0)  92 (74.8)  2.58 (1.34)  92 (74.8)  64 (52.0)  32 (26.0)  74 (60.2)  31 (25.2)  8 (6.5)  3 (2.4)  3 (2.4)  56 (45.5)  47 (38.2)  10 (8.1)  40 (32.5)  37 (30.1)  40 (32.5)  13 (10.6)  54 (43.9)  9 (7.3)  4 (3.3)  8 (6.5)    8.37 (5.09)  5.03 (2.58)  7.89 (1.59)  2.51 (1.65)  4.11 (2.30)  2.55 (1.78)  16.50 (2.99)  17.63 (3.61)  60 (48.8)  24 (19.5)  62 (50.4)  22 (17.9)  78 (63.4)  43 (35.0)  21 (17.1)  37 (30.1)  51 (41.5)  38 (30.9)  14 (11.4)  47 (38.2)  75 (61.0)  38 (30.9)  44 (35.8)  16 (13.0) | 12.63 (0.62)  46 (37.4)  4 (3.3)  36 (29.3)  43 (35.0)  92 (74.8)  2.58 (1.34)  92 (74.8)  64 (52.0)  32 (26.0)  73 (59.4)  31 (25.2)  8 (6.5)  3 (2.4)  3 (2.4)  56 (45.5)  47 (38.2)  10 (8.1)  40 (32.5)  37 (30.1)  40 (32.5)  13 (10.6)  54 (43.9)  9 (7.3)  4 (3.3)  8 (6.5)    8.28 (4.96)  5.03 (2.58)  7.89 (1.58)  2.51 (1.65)  4.11 (2.30)  2.55 (1.78)  16.49 (2.97)  17.63 (3.61)  60 (48.8)  24 (19.5)  62 (50.4)  21 (17.1)  79 (64.2)  43 (35.0)  21 (17.1)  37 (30.1)  51 (41.5)  38 (30.9)  14 (11.4)  47 (38.2)  75 (61.0)  37 (30.1)  44 (35.8)  16 (13.0) | 12.63 (0.62)  46 (37.4)  4 (3.3)  37 (30.1)  43 (35.0)  92 (74.8)  2.58 (1.34)  92 (74.8)  64 (52.0)  32 (26.0)  73 (59.4)  31 (25.2)  8 (6.5)  3 (2.4)  3 (2.4)  56 (45.5)  47 (38.2)  10 (8.1)  40 (32.5)  37 (30.1)  39 (31.7)  13 (10.6)  54 (43.9)  10 (8.1)  4 (3.3)  9 (7.3)    8.26 (5.00)  5.03 (2.58)  7.91 (1.58)  2.51 (1.65)  4.11 (2.30)  2.55 (1.78)  16.43 (3.01)  11.65 (3.54)  60 (48.8)  24 (19.5)  62 (50.4)  21 (17.1)  78 (63.4)  43 (35.0)  21 (17.1)  37 (30.1)  51 (41.5)  38 (30.9)  14 (11.4)  47 (38.2)  75 (61.0)  38 (30.9)  44 (35.8)  17 (13.8) |

Abbreviations:

AVH – Auditory Verbal Hallucinations

N – Number of participants

PQ-16 – 16 item Prodromal Questionnaire

RSE – Rosenberg Self-Esteem Scale

SAHA – Social And Health Assessment

SD – Standard Deviation

SDQ – Strengths and Difficulties Questionnaire (for age 4-17 years)

***Supplement 2***

Characteristics of the group of participants with persistence of distressing AVH (N=43).

| Characteristics | 1 | 2 | 3 | 4 | 5 | 6 | 7 | 8 | 9 | 10 |
| --- | --- | --- | --- | --- | --- | --- | --- | --- | --- | --- |
| Age (years) *mean (SD)*  Male gender *N (%)*  Not born in The Netherlands *N (%)*  Migrant-Dutch ethnicity *N (%)*  Religious *N (%)*  Living in a large town (>110.000 residents) *N (%)*  Number of children in the family (including participant) *mean (SD)*  High socioeconomic status of the family *N (%)*  High education level *N (%)*  School grade repetition *N (%)*  Moved houses (ever) *N (%)*  Parents divorced *N (%)*  Parents divorced in past year *N (%)*  Parent deceased *N (%)*  Parent deceased in past year *N (%)*  Traumatic experiences: *N (%)*  - Been scared that an acquaintance would be killed or seriously injured  - Attacked, beaten up or threatened  - Sexually assaulted  - Been in or near a serious accident  - Seen or heard someone being killed, passed away or seriously injured  - Been scared by seeing a deceased body  Been discriminated on skin colour, ethnicity or religion in past year *N (%)*  Substance use: *N (%)*  - alcohol (ever)  - been drunk in past year  - been in a fight while drinking alcohol in past year  - cannabis (ever)  SAHA somatic complaints (range: 0-20) *mean (SD)*  SDQ scales: *mean (SD)*  - Hyperactivity (range: 0-10)  - Prosocial behaviour (range: 0-10)  - Conduct problems (range: 0-10)  - Emotional problems (range: 0-10)  - Peer problems (range: 0-10)  RSE (self-esteem) total score (range: 0-30) *mean (SD)*  Mastery total score (range: 0-26) *mean (SD)*  High peer status *N (%)*  PQ-16 psychotic experiences: *N (%)*  - I feel uninterested in the things I used to enjoy.  - I often live through events exactly as they happened before.  - I sometimes smell or taste things that other people do not notice.  - I often hear unusual sounds in my ears.  - I have been confused at times whether something I experienced was real or imaginary.  - When I look at a person, or at myself in a mirror, I have seen the face change right before me.  - I get extremely anxious when meeting people for the first time.  - I have seen things that other people cannot or do not see.  - My thoughts are sometimes so strong that I can almost hear them.  - I sometimes see special meanings in advertisements, shop windows, or in the way things are arranged around me.  - I have felt not in control of my own ideas or thoughts.  - I sometimes feel suddenly distracted by distant sounds that I am not normally aware of.  - I often feel that others have it in for me.  - I have had the sense that a person or force is around me, even though I did not see anyone.  - I feel that parts of my body have changed in some way, or that parts of my body are working differently. | 12.56 (0.67)  15 (34.9)  1 (2.3)  14 (32.6)  15 (34.9)  32 (74.4)  2.60 (1.50)  29 (67.4)  25 (58.1)  6 (14.0)  26 (60.5)  11 (25.6)  6 (14.0)  1 (2.3)  2 (4.7)  24 (55.8)  18 (41.9)  6 (14.0)  14 (32.6)  14 (32.6)  18 (41.9)  4 (9.3)  23 (53.5)  6 (14.0)  2 (4.7)  6 (14.0)  8.79 (5.28)  4.63 (2.96)  8.21 (1.74)  2.28 (1.55)  4.07 (2.20)  2.40 (1.81)  16.16 (2.39)  17.72 (3.85)  22 (51.2)  9 (20.9)  16 (37.2)  8 (18.6)  26 (60.5)  15 (34.9)  5 (11.6)  9 (20.9)  20 (46.5)  14 (32.6)  4 (9.3)  16 (37.2)  28 (65.1)  7 (16.3)  18 (41.9)  2 (4.7) | 12.56 (0.67)  15 (34.9)  1 (2.3)  15 (34.9)  15 (34.9)  32 (74.4)  2.60 (1.50)  29 (67.4)  25 (58.1)  6 (14.0)  26 (60.5)  11 (25.6)  6 (14.0)  1 (2.3)  2 (4.7)  24 (55.8)  18 (41.9)  6 (14.0)  14 (32.6)  14 (32.6)  18 (41.9)  4 (9.3)  23 (53.5)  6 (14.0)  2 (4.7)  6 (14.0)  8.84 (5.25)  4.63 (2.96)  8.21 (1.74)  2.28 (1.55)  4.07 (2.20)  2.40 (1.81)  16.14 (2.39)  17.72 (3.85)  22 (51.2)  9 (20.9)  16 (37.2)  7 (16.3)  26 (60.5)  15 (34.9)  5 (11.6)  9 (20.9)  20 (46.5)  14 (32.6)  4 (9.3)  16 (37.2)  27 (62.8)  7 (16.3)  18 (41.9)  2 (4.7) | 12.56 (0.67)  15 (34.9)  1 (2.3)  15 (34.9)  15 (34.9)  32 (74.4)  2.60 (1.50)  29 (67.4)  25 (58.1)  6 (14.0)  26 (60.5)  11 (25.6)  6 (14.0)  1 (2.3)  2 (4.7)  24 (55.8)  18 (41.9)  6 (14.0)  14 (32.6)  14 (32.6)  18 (41.9)  4 (9.3)  23 (53.5)  6 (14.0)  2 (4.7)  6 (14.0)  8.95 (5.33)  4.63 (2.96)  78.21 (1.74)  2.28 (1.55)  4.07 (2.20)  2.40 (1.81)  16.16 (2.39)  17.72 (3.85)  22 (51.2)  9 (20.9)  16 (37.2)  6 (14.0)  27 (62.8)  15 (34.9)  5 (11.6)  9 (20.9)  20 (46.5)  14 (32.6)  4 (9.3)  16 (37.2)  27 (62.8)  7 (16.3)  18 (41.9)  2 (4.7) | 12.56 (0.67)  15 (34.9)  1 (2.3)  14 (32.6)  15 (34.9)  32 (74.4)  2.60 (1.50)  29 (67.4)  25 (58.1)  6 (14.0)  27 (62.8)  10 (23.3)  6 (14.0)  1 (2.3)  2 (4.7)  24 (55.8)  18 (41.9)  6 (14.0)  14 (32.6)  14 (32.6)  18 (41.9)  4 (9.3)  23 (53.5)  6 (14.0)  2 (4.7)  6 (14.0)  8.86 (5.26)  4.63 (2.96)  8.21 (1.74)  2.28 (1.55)  4.07 (2.20)  2.40 (1.81)  16.14 (2.39)  17.72 (3.85)  22 (51.2)  9 (20.9)  16 (37.2)  6 (14.0)  26 (60.5)  15 (34.9)  5 (11.6)  9 (20.9)  20 (46.5)  14 (32.6)  4 (9.3)  16 (37.2)  27 (62.8)  7 (16.3)  18 (41.9)  2 (4.7) | 12.56 (0.67)  15 (34.9)  1 (2.3)  14 (32.6)  15 (34.9)  32 (74.4)  2.60 (1.50)  29 (67.4)  25 (58.1)  6 (14.0)  26 (60.5)  11 (25.6)  6 (14.0)  1 (2.3)  2 (4.7)  24 (55.8)  18 (41.9)  6 (14.0)  14 (32.6)  14 (32.6)  18 (41.9)  4 (9.3)  23 (53.5)  6 (14.0)  2 (4.7)  6 (14.0)  8.93 (5.36)  4.63 (2.96)  8.21 (1.74)  2.28 (1.55)  4.07 (2.20)  2.40 (1.81)  16.19 (2.40)  17.72 (3.85)  22 (51.2)  9 (20.9)  16 (37.2)  6 (14.0)  26 (60.5)  15 (34.9)  5 (11.6)  9 (20.9)  20 (46.5)  14 (32.6)  4 (9.3)  16 (37.2)  28 (65.1)  7 (16.3)  18 (41.9)  2 (4.7) | 12.56 (0.67)  15 (34.9)  1 (2.3)  14 (32.6)  15 (34.9)  32 (74.4)  2.60 (1.50)  29 (67.4)  25 (58.1)  6 (14.0)  26 (60.5)  11 (25.6)  6 (14.0)  1 (2.3)  2 (4.7)  24 (55.8)  18 (41.9)  6 (14.0)  14 (32.6)  14 (32.6)  18 (41.9)  4 (9.3)  23 (53.5)  6 (14.0)  2 (4.7)  6 (14.0)  8.93 (5.31)  4.63 (2.96)  8.21 (1.74)  2.28 (1.55)  4.07 (2.20)  2.40 (1.81)  16.21 (2.43)  17.72 (3.85)  22 (51.2)  9 (20.9)  16 (37.2)  7 (16.3)  26 (60.5)  15 (34.9)  5 (11.6)  9 (20.9)  20 (46.5)  14 (32.6)  4 (9.3)  16 (37.2)  27 (62.8)  7 (16.3)  18 (41.9)  2 (4.7) | 12.56 (0.67)  15 (34.9)  1 (2.3)  14 (32.6)  15 (34.9)  32 (74.4)  2.60 (1.50)  29 (67.4)  25 (58.1)  6 (14.0)  26 (60.5)  11 (25.6)  6 (14.0)  1 (2.3)  2 (4.7)  24 (55.8)  18 (41.9)  6 (14.0)  14 (32.6)  14 (32.6)  18 (41.9)  4 (9.3)  23 (53.5)  6 (14.0)  2 (4.7)  6 (14.0)  8.88 (5.27)  4.63 (2.96)  8.21 (1.74)  2.28 (1.55)  4.07 (2.20)  2.40 (1.81)  16.07 (2.43)  17.72 (3.85)  22 (51.2)  9 (20.9)  16 (37.2)  7 (16.3)  26 (60.5)  15 (34.9)  5 (11.6)  9 (20.9)  20 (46.5)  14 (32.6)  4 (9.3)  16 (37.2)  27 (62.8)  7 (16.3)  18 (41.9)  2 (4.7) | 12.56 (0.67)  15 (34.9)  1 (2.3)  14 (32.6)  15 (34.9)  32 (74.4)  2.60 (1.50)  29 (67.4)  25 (58.1)  6 (14.0)  26 (60.5)  11 (25.6)  6 (14.0)  1 (2.3)  2 (4.7)  24 (55.8)  18 (41.9)  6 (14.0)  14 (32.6)  14 (32.6)  18 (41.9)  4 (9.3)  23 (53.5)  6 (14.0)  2 (4.7)  6 (14.0)  8.70 (5.21)  4.63 (2.96)  8.21 (1.74)  2.28 (1.55)  4.07 (2.20)  2.40 (1.81)  16.23 (2.46)  17.72 (3.85)  22 (51.2)  9 (20.9)  16 (37.2)  7 (16.3)  26 (60.5)  15 (34.9)  5 (11.6)  9 (20.9)  20 (46.5)  14 (32.6)  4 (9.3)  16 (37.2)  27 (62.8)  7 (16.3)  18 (41.9)  2 (4.7) | 12.56 (0.67)  15 (34.9)  1 (2.3)  14 (32.6)  15 (34.9)  32 (74.4)  2.60 (1.50)  29 (67.4)  25 (58.1)  6 (14.0)  26 (60.5)  11 (25.6)  6 (14.0)  1 (2.3)  2 (4.7)  24 (55.8)  18 (41.9)  6 (14.0)  14 (32.6)  14 (32.6)  18 (41.9)  4 (9.3)  23 (53.5)  6 (14.0)  2 (4.7)  6 (14.0)  8.81 (5.34)  4.63 (2.96)  8.21 (1.74)  2.28 (1.55)  4.07 (2.20)  2.40 (1.81)  16.16 (2.39)  17.72 (3.85)  22 (51.2)  9 (20.9)  16 (37.2)  7 (16.3)  26 (60.5)  15 (34.9)  5 (11.6)  9 (20.9)  20 (46.5)  14 (32.6)  4 (9.3)  16 (37.2)  27 (62.8)  7 (16.3)  18 (41.9)  2 (4.7) | 12.56 (0.67)  15 (34.9)  1 (2.3)  15 (34.9)  15 (34.9)  32 (74.4)  2.60 (1.50)  29 (67.4)  25 (58.1)  6 (14.0)  26 (60.5)  11 (25.6)  6 (14.0)  1 (2.3)  2 (4.7)  24 (55.8)  18 (41.9)  6 (14.0)  14 (32.6)  14 (32.6)  18 (41.9)  4 (9.3)  23 (53.5)  6 (14.0)  2 (4.7)  6 (14.0)  8.77 (5.25)  4.63 (2.96)  8.21 (1.74)  2.28 (1.55)  4.07 (2.20)  2.40 (1.81)  16.05 (2.47)  17.72 (3.85)  22 (51.2)  9 (20.9)  16 (37.2)  7 (16.3)  26 (60.5)  15 (34.9)  5 (11.6)  9 (20.9)  20 (46.5)  14 (32.6)  4 (9.3)  16 (37.2)  27 (62.8)  7 (16.3)  18 (41.9)  2 (4.7) |

Abbreviations:

AVH – Auditory Verbal Hallucinations

N – Number of participants

PQ-16 – 16 item Prodromal Questionnaire

RSE – Rosenberg Self-Esteem Scale

SAHA – Social And Health Assessment

SD – Standard Deviation

SDQ – Strengths and Difficulties Questionnaire (for age 4-17 years)

***Supplement 3***

Characteristics of the group of participants with discontinuation of distressing AVH (N=80).

| Characteristics | 1 | 2 | 3 | 4 | 5 | 6 | 7 | 8 | 9 | 10 |
| --- | --- | --- | --- | --- | --- | --- | --- | --- | --- | --- |
| Age (years) *mean (SD)*  Male gender *N (%)*  Not born in The Netherlands *N (%)*  Migrant-Dutch ethnicity *N (%)*  Religious *N (%)*  Living in a large town (>110.000 residents) *N (%)*  Number of children in the family (including participant) *mean (SD)*  High socioeconomic status of the family *N (%)*  High education level *N (%)*  School grade repetition *N (%)*  Moved houses (ever) *N (%)*  Parents divorced *N (%)*  Parents divorced in past year *N (%)*  Parent deceased *N (%)*  Parent deceased in past year *N (%)*  Traumatic experiences: *N (%)*  - Been scared that an acquaintance would be killed or seriously injured  - Attacked, beaten up or threatened  - Sexually assaulted  - Been in or near a serious accident  - Seen or heard someone being killed, passed away or seriously injured  - Been scared by seeing a deceased body  Been discriminated on skin colour, ethnicity or religion in past year *N (%)*  Substance use: *N (%)*  - alcohol (ever)  - been drunk in past year  - been in a fight while drinking alcohol in past year  - cannabis (ever)  SAHA somatic complaints (range: 0-20) *mean (SD)*  SDQ scales: *mean (SD)*  - Hyperactivity (range: 0-10)  - Prosocial behaviour (range: 0-10)  - Conduct problems (range: 0-10)  - Emotional problems (range: 0-10)  - Peer problems (range: 0-10)  RSE (self-esteem) total score (range: 0-30) *mean (SD)*  Mastery total score (range: 0-26) *mean (SD)*  High peer status *N (%)*  PQ-16 psychotic experiences: *N (%)*  - I feel uninterested in the things I used to enjoy.  - I often live through events exactly as they happened before.  - I sometimes smell or taste things that other people do not notice.  - I often hear unusual sounds in my ears.  - I have been confused at times whether something I experienced was real or imaginary.  - When I look at a person, or at myself in a mirror, I have seen the face change right before me.  - I get extremely anxious when meeting people for the first time.  - I have seen things that other people cannot or do not see.  - My thoughts are sometimes so strong that I can almost hear them.  - I sometimes see special meanings in advertisements, shop windows, or in the way things are arranged around me.  - I have felt not in control of my own ideas or thoughts.  - I sometimes feel suddenly distracted by distant sounds that I am not normally aware of.  - I often feel that others have it in for me.  - I have had the sense that a person or force is around me, even though I did not see anyone.  - I feel that parts of my body have changed in some way, or that parts of my body are working differently. | 12.66 (0.59)  31 (38.8)  3 (3.8)  22 (27.5)  28 (35.0)  60 (75.0)  2.56 (1.26)  63 (78.8)  39 (48.8)  26 (32.5)  48 (60.0)  20 (25.0)  2 (2.5)  2 (2.5)  1 (1.3)  32 (40.0)  29 (36.3)  4 (5.0)  26 (32.5)  23 (28.8)  22 (27.5)  9 (11.3)  31 (38.8)  3 (3.8)  2 (2.5)  2 (2.5)  8.03 (4.80)  5.25 (2.34)  7.70 (1.50)  2.64 (1.70)  4.14 (2.36)  2.64 (1.77)  16.73 (3.22)  17.57 (3.50)  38 (47.5)  15 (18.8)  46 (57.5)  14 (17.5)  52 (65.0)  28 (35.0)  16 (20.0)  28 (35.0)  31 (38.8)  24 (30.0)  10 (12.5)  31 (38.8)  48 (60.0)  30 (37.5)  27 (33.8)  14 (17.5) | 12.66 (0.59)  31 (38.8)  3 (3.8)  23 (28.8)  28 (35.0)  60 (75.0)  2.56 (1.26)  63 (78.8)  39 (48.8)  26 (32.5)  48 (60.0)  20 (25.0)  2 (2.5)  2 (2.5)  1 (1.3)  32 (40.0)  29 (36.3)  4 (5.0)  26 (32.5)  23 (28.8)  22 (27.5)  9 (11.3)  31 (38.8)  3 (3.8)  2 (2.5)  3 (3.8)  7.83 (4.79)  5.25 (2.34)  7.76 (1.49)  2.64 (1.70)  4.14 (2.36)  2.64 (1.77)  16.69 (3.27)  17.57 (3.50)  38 (47.5)  15 (18.8)  46 (57.5)  14 (17.5)  53 (66.3)  28 (35.0)  16 (20.0)  28 (35.0)  31 (38.8)  24 (30.0)  10 (12.5)  31 (38.8)  48 (60.0)  31 (38.8)  26 (32.5)  14 (17.5) | 12.66 (0.59)  31 (38.8)  3 (3.8)  22 (27.5)  28 (35.0)  60 (75.0)  2.56 (1.26)  63 (78.8)  39 (48.8)  26 (32.5)  47 (58.8)  20 (25.0)  2 (2.5)  2 (2.5)  1 (1.3)  32 (40.0)  29 (36.3)  4 (5.0)  26 (32.5)  23 (28.8)  22 (27.5)  9 (11.3)  31 (38.8)  3 (3.8)  2 (2.5)  3 (3.8)  8.18 (4.91)  5.25 (2.34)  7.71 (1.49)  2.64 (1.70)  4.14 (2.36)  2.64 (1.77)  16.69 (3.22)  17.57 (3.50)  38 (47.5)  15 (18.8)  46 (57.5)  14 (17.5)  53 (66.3)  28 (35.0)  16 (20.0)  28 (35.0)  31 (38.8)  24 (30.0)  10 (12.5)  31 (38.8)  48 (60.0)  30 (37.5)  26 (32.5)  15 (18.8) | 12.66 (0.59)  31 (38.8)  3 (3.8)  22 (27.5)  28 (35.0)  60 (75.0)  2.56 (1.26)  63 (78.8)  39 (48.8)  26 (32.5)  47 (58.8)  20 (25.0)  2 (2.5)  2 (2.5)  1 (1.3)  32 (40.0)  29 (36.3)  4 (5.0)  26 (32.5)  23 (28.8)  22 (27.5)  9 (11.3)  31 (38.8)  4 (5.0)  2 (2.5)  3 (3.8)  7.90 (4.73)  5.25 (2.34)  7.72 (1.48)  2.64 (1.70)  4.14 (2.36)  2.64 (1.77)  16.73 (3.21)  17.57 (3.50)  38 (47.5)  15 (18.8)  46 (57.5)  12 (15.0)  53 (66.3)  28 (35.0)  17 (21.3)  28 (35.0)  31 (38.8)  24 (30.0)  10 (12.5)  31 (38.8)  48 (60.0)  30 (37.5)  26 (32.5)  14 (17.5) | 12.66 (0.59)  31 (38.8)  3 (3.8)  22 (27.5)  28 (35.0)  60 (75.0)  2.56 (1.26)  63 (78.8)  39 (48.8)  26 (32.5)  48 (60.0)  20 (25.0)  2 (2.5)  2 (2.5)  1 (1.3)  32 (40.0)  29 (36.3)  4 (5.0)  26 (32.5)  23 (28.8)  22 (27.5)  9 (11.3)  31 (38.8)  3 (3.8)  2 (2.5)  3 (3.8)  8.14 (4.96)  5.25 (2.34)  7.74 (1.47)  2.64 (1.70)  4.14 (2.36)  2.64 (1.77)  16.65 (3.24)  17.57 (3.50)  38 (47.5)  15 (18.8)  46 (57.5)  13 (16.3)  53 (66.3)  28 (35.0)  16 (20.0)  28 (35.0)  31 (38.8)  24 (30.0)  10 (12.5)  31 (38.8)  48 (60.0)  30 (37.5)  26 (32.5)  14 (17.5) | 12.66 (0.59)  31 (38.8)  3 (3.8)  22 (27.5)  28 (35.0)  60 (75.0)  2.56 (1.26)  63 (78.8)  39 (48.8)  26 (32.5)  46 (57.5)  20 (25.0)  2 (2.5)  2 (2.5)  1 (1.3)  32 (40.0)  29 (36.3)  4 (5.0)  26 (32.5)  23 (28.8)  21 (26.3)  9 (11.3)  31 (38.8)  4 (5.0)  3 (3.8)  3 (3.8)  8.16 (4.89)  5.25 (2.34)  7.74 (1.47)  2.64 (1.70)  4.14 (2.36)  2.64 (1.77)  16.73 (3.26)  17.57 (3.50)  38 (47.5)  15 (18.8)  46 (57.5)  14 (17.5)  53 (66.3)  28 (35.0)  16 (20.0)  28 (35.0)  31 (38.8)  24 (30.0)  10 (12.5)  31 (38.8)  48 (60.0)  30 (37.5)  26 (32.5)  15 (18.8) | 12.66 (0.59)  31 (38.8)  3 (3.8)  22 (27.5)  28 (35.0)  60 (75.0)  2.56 (1.26)  63 (78.8)  39 (48.8)  26 (32.5)  46 (57.5)  20 (25.0)  2 (2.5)  2 (2.5)  1 (1.3)  32 (40.0)  29 (36.3)  4 (5.0)  26 (32.5)  23 (28.8)  22 (27.5)  9 (11.3)  31 (38.8)  4 (5.0)  2 (2.5)  2 (2.5)  7.91 (4.72)  5.25 (2.34)  7.74 (1.47)  2.64 (1.70)  4.14 (2.36)  2.64 (1.77)  16.60 (3.38)  17.57 (3.50)  38 (47.5)  15 (18.8)  46 (57.5)  14 (17.5)  53 (66.3)  28 (35.0)  16 (20.0)  28 (35.0)  31 (38.8)  24 (30.0)  10 (12.5)  31 (38.8)  48 (60.0)  29 (36.3)  26 (32.5)  14 (17.5) | 12.66 (0.59)  31 (38.8)  3 (3.8)  22 (27.5)  28 (35.0)  60 (75.0)  2.56 (1.26)  63 (78.8)  39 (48.8)  26 (32.5)  48 (60.0)  20 (25.0)  2 (2.5)  2 (2.5)  1 (1.3)  32 (40.0)  29 (36.3)  4 (5.0)  26 (32.5)  23 (28.8)  22 (27.5)  9 (11.3)  31 (38.8)  3 (3.8)  2 (2.5)  2 (2.5)  8.20 (5.05)  5.25 (2.34)  7.71 (1.49)  2.64 (1.70)  4.14 (2.36)  2.64 (1.77)  16.65 (3.25)  17.57 (3.50)  38 (47.5)  15 (18.8)  46 (57.5)  15 (18.8)  52 (65.0)  28 (35.0)  16 (20.0)  28 (35.0)  31 (38.8)  24 (30.0)  10 (12.5)  31 (38.8)  48 (60.0)  31 (38.8)  26 (32.5)  14 (17.5) | 12.66 (0.59)  31 (38.8)  3 (3.8)  22 (27.5)  28 (35.0)  60 (75.0)  2.56 (1.26)  63 (78.8)  39 (48.8)  26 (32.5)  47 (58.8)  20 (25.0)  2 (2.5)  2 (2.5)  1 (1.3)  32 (40.0)  29 (36.3)  4 (5.0)  26 (32.5)  23 (28.8)  22 (27.5)  9 (11.3)  31 (38.8)  3 (3.8)  2 (2.5)  2 (2.5)  7.99 (4.75)  5.25 (2.34)  7.72 (1.48)  2.64 (1.70)  4.14 (2.36)  2.64 (1.77)  16.66 (3.24)  17.57 (3.50)  38 (47.5)  15 (18.8)  46 (57.5)  14 (17.5)  53 (66.3)  28 (35.0)  16 (20.0)  28 (35.0)  31 (38.8)  24 (30.0)  10 (12.5)  31 (38.8)  48 (60.0)  30 (37.5)  26 (32.5)  14 (17.5) | 12.66 (0.59)  31 (38.8)  3 (3.8)  22 (27.5)  28 (35.0)  60 (75.0)  2.56 (1.26)  63 (78.8)  39 (48.8)  26 (32.5)  47 (58.8)  20 (25.0)  2 (2.5)  2 (2.5)  1 (1.3)  32 (40.0)  29 (36.3)  4 (5.0)  26 (32.5)  23 (28.8)  21 (26.3)  9 (11.3)  31 (38.8)  4 (5.0)  2 (2.5)  3 (3.8)  7.99 (4.87)  5.25 (2.34)  7.75 (1.48)  2.64 (1.70)  4.14 (2.36)  2.64 (1.77)  16.64 (3.26)  17.57 (3.50)  38 (47.5)  15 (18.8)  46 (57.5)  14 (17.5)  52 (65.0)  28 (35.0)  16 (20.0)  28 (35.0)  31 (38.8)  24 (30.0)  10 (12.5)  31 (38.8)  48 (60.0)  31 (38.8)  26 (32.5)  15 (18.8) |

Abbreviations:

AVH – Auditory Verbal Hallucinations

N – Number of participants

PQ-16 – 16 item Prodromal Questionnaire

RSE – Rosenberg Self-Esteem Scale

SAHA – Social And Health Assessment

SD – Standard Deviation

SDQ – Strengths and Difficulties Questionnaire (for age 4-17 years)
